# Retraction Note: Laparoscopic repair of vesicovaginal fistulae with a transperitoneal approach at Universitas Gadjah Mada Urological Institute: a case report

**DOI:** 10.1186/s13256-022-03569-3

**Published:** 2022-08-22

**Authors:** Indrawarman Soeroharjo, Said Alfin Khalilullah, Raden Danarto, Prahara Yuri

**Affiliations:** Division of Urology, Department of Surgery, Faculty of Medicine, Universitas Gadjah Mada/Dr. Sardjito Hospital, Yogyakarta, 55281 Indonesia

## Retraction Note to: Journal of Medical Case Reports (2018) 12:47 https://doi.org/10.1186/s13256-018-1582-6

The Editor-in-Chief has retracted this case report because Figure 3a and Figure 3b are the same as Figure 2 and Figure 3 respectively in [1] which describes a different patient, and the text of the background and discussion sections shows overlap with [1–4]. Said Alfin Khalilullah and Prahara Yuri agree with this retraction. Indrawarman Soeroharjo and Raden Danarto have not responded to correspondence from the publisher about this retraction.
